# Pancreatoscopy-assisted balloon dilation under direct vision in a porcine model

**DOI:** 10.1055/a-2462-1408

**Published:** 2024-11-18

**Authors:** Wengang Zhang, Haiyang Li, Zhenyu Liu, Jiafeng Wang, Qingzhen Wu, Ningli Chai, Enqiang Linghu

**Affiliations:** 1 Gastroenterology, The First Medical Center of Chinese PLA General Hospital, Beijing, China

Chronic pancreatitis is an inflammatory condition characterized by irreversible morphological changes in the pancreas, which invariably leads to pancreatic duct (PD) strictures 1 . Currently, PD strictures are typically managed with wire-guided balloon or bougie dilation under X-ray guidance 2 3 . However, these methods carry a risk of PD perforation.

This study introduces a novel approach involving pancreatoscopy-assisted balloon dilation under direct visualization in a porcine model. First, PD intubation was performed, and a cholangioscope (8 F; eyeMAX; Micro-Tech, Nanjing, China) was advanced into the PD in the porcine model ( Fig. 1 , Fig. 2 ). A novel balloon, designed to pass through the working channel of the cholangioscope, was then inserted into the PD ( Fig. 3 , Fig. 4 ). Subsequently, balloon dilation was conducted under direct visualization ( Fig. 5 , Video 1 ). Finally, the balloon was deflated and withdrawn from the PD. No serious adverse events were observed during the 1-week follow-up period.

**Fig. 1:**
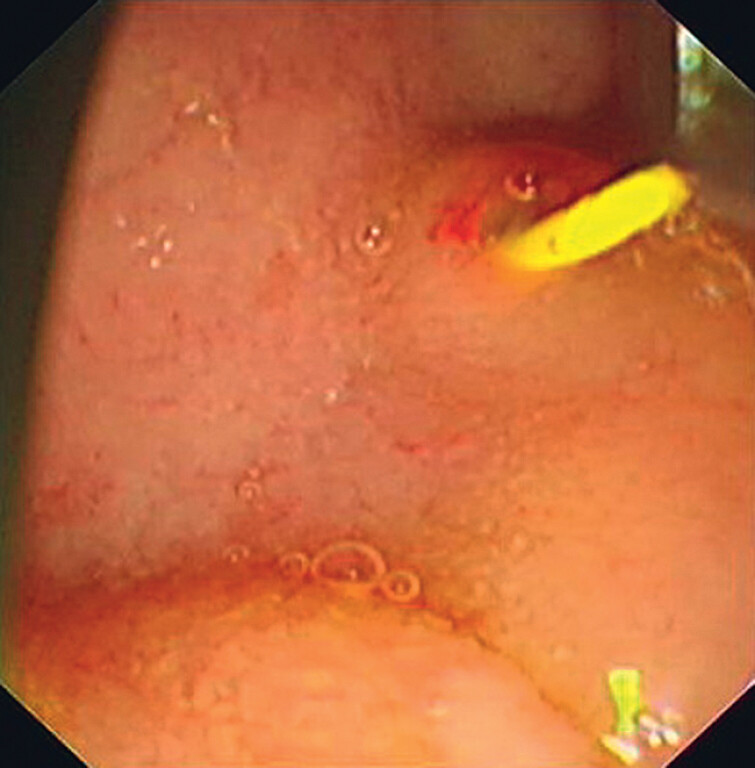
Pancreatic duct intubation was conducted in a porcine model.

**Fig. 2:**
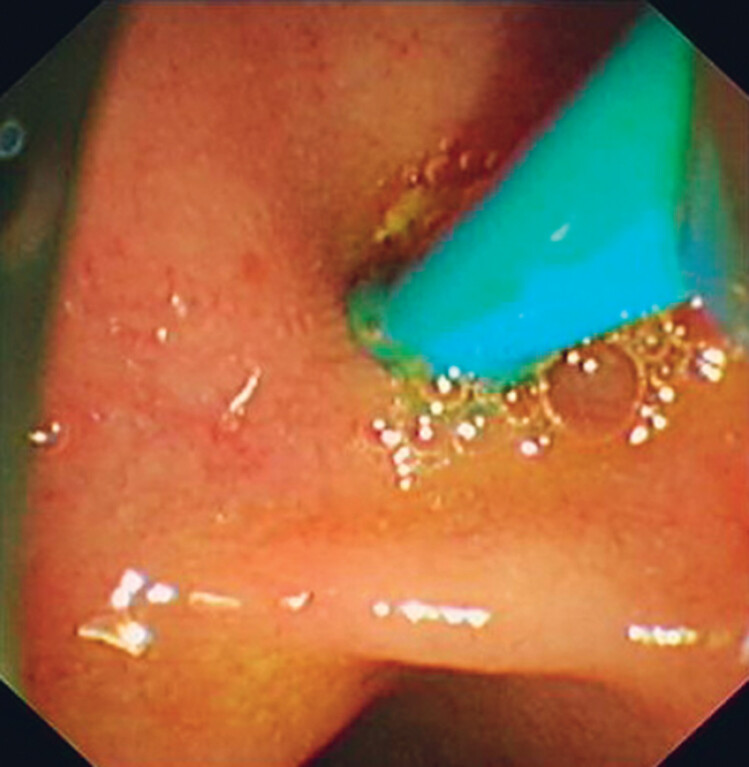
The cholangioscope (8 F; eyeMAX; Micro-Tech, Nanjing, China) was inserted into the pancreatic duct.

**Fig. 3:**
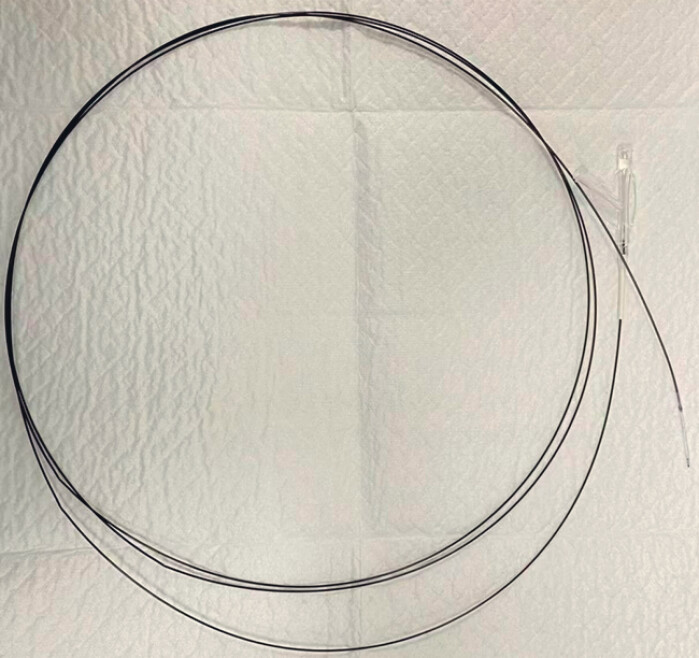
The novel balloon can pass through the working channel of the cholangioscope.

**Fig. 4:**
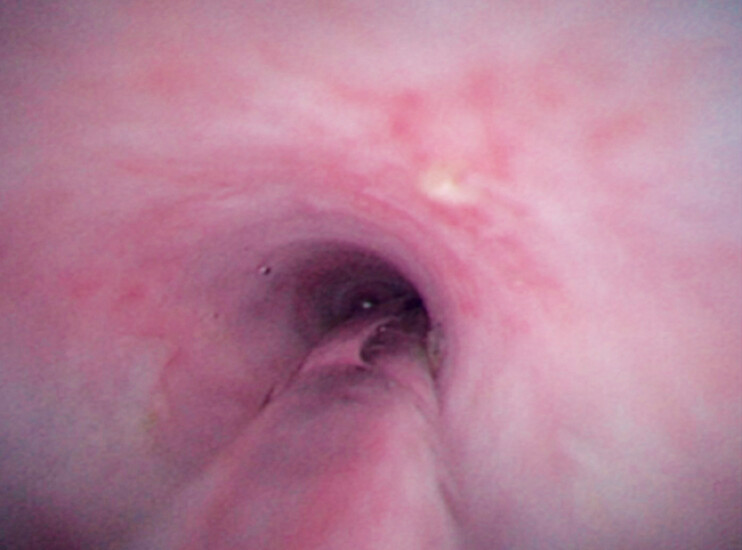
The novel balloon was inserted into the pancreatic duct.

**Fig. 5:**
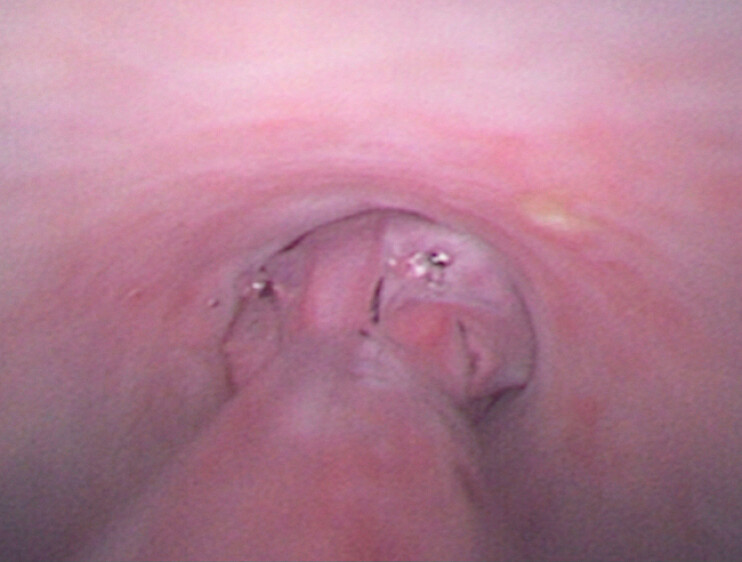
Balloon dilation was performed under direct visualization.

Pancreatoscopy-assisted balloon dilation was performed in a porcine model.Video 1

The primary advantage of this technique over traditional wire-guided balloon or bougie dilation under X-ray is that it allows the endoscopist to observe the degree of dilation in a timely manner. Theoretically, this technique could also be applied to the biliary duct or cystic duct. Notably, our team has been exploring cholangioscopy-assisted extraction of gallstones 4 . For patients with a narrow cystic duct, this approach can often achieve optimal results. We propose the term cholangiopancreatoscopy-assisted balloon dilation (CABD) for this technique.

This study preliminary demonstrates the feasibility of pancreatoscopy-assisted balloon dilation in a porcine model. Future applications of this technique may offer significant benefits for patients with biliary duct or PD strictures.

Endoscopy_UCTN_Code_TTT_1AS_2AI

## Notes

Endoscopy E-Videos https://eref.thieme.de/e-videos
*E-Videos* is an open access online section of the journal *Endoscopy* , reporting on interesting cases and new techniques in gastroenterological endoscopy. All papers include a high-quality video and are published with a Creative Commons CC-BY license. Endoscopy E-Videos qualify for HINARI discounts and waivers and eligibility is automatically checked during the submission process. We grant 100% waivers to articles whose corresponding authors are based in Group A countries and 50% waivers to those who are based in Group B countries as classified by Research4Life (see: https://www.research4life.org/access/eligibility/ ). This section has its own submission website at https://mc.manuscriptcentral.com/e-videos .
